# The Surgical Idea in Obstetrics

**Published:** 1909-03

**Authors:** William Mortimer Brown

**Affiliations:** Rochester, N. Y.


### The Surgical Idea in Obstetrics

BY WILLIAM MORTIMER BROWN, M. D., Rochester, N. Y.

IN choosing the subject of this paper I had in mind to discuss the question of surgical principles in their relation to obstetrics believing, as we must at the present time, that good obstetrical work is dependent on a thorough grounding in the basic principles of surgery. My remarks must necessarily be brief and it is my intention to take up the discussion of special surgical operations in the obstetrical field somewhat superficially and more as a means of illustration and to emphasize the value of good surgical judgment and training in the practice of midwifery.[1]

Realising that the specialist in obstetrics is at best only a consultant and should not be in active competition for normal cases as it is to the general practitioner that at least the normal cases belong and especially in isolated communities, it follows that any advancement or bettering of methods which will result in less morbidity and a lower mortality rate must come from those men, who will avail themselves of the knowledge gained by men who are giving this work definite study and who will apply that knowledge in their daily practice.

Thus having seen that it is the general practitioner, the family physician, the busy man who works all day and often all night, who does the obstetrical work in a community, we realise that if he is any one thing more than another he is an internist rather than a surgeon, consequently the conduct of an ordinary confinement case is usually a surgical procèdure done by a medical man if indeed he has not been a midwife.

It is easy, I think, to see why this condition has prevailed. In fact it is the logical development of the conditions along the

---

1. Read at the forty-first annual meeting of the Medical Association of Central New York, held at Buffalo. October 27, 1908.

lines of our past knowledge, false as we now know much of that knowledge to have been.

Biologically and physiologically speaking, a woman needs no outside aid after pregnancy is established, to reproduce her kind. Primarily, she is able to develop and deliver her offspring.

In primitive times and later under conditions simulating the primitive, and even now in isolated sections where conditions prevail that are of a more or less primitive character, the delivery of woman was or is a matter to which she must attend herself; but in the gradual evolution of social conditions the woman has grown more and more to rely upon outside aid in her accouchement.

From being doomed to solitude during her labor like the Huskie woman of the north, who leaves her hut at the beginning of labor and digs for herself a cave in the snow where she hides until the child is born, the woman in travail was first given the company of her kind with their sympathy and simple ministrations. From this time on the employment of some form of outside aid in various ways during a confinement has progressed through many stages from the members of the family to the officious next door neighbor, to the woman in the vicinity who has had a child and knows it all, to some woman who has seen so many cases that she is able to tell every one else all about it but who usually sits by and does nothing, to the midwife who by virtue of some training was considered to be of great value but whose value was destroyed by the infections she spread in her wake, to the time when the doctor came into the field .

During the time when the woman was her own accoucheur sepsis and accidents of labor were practically unknown, but in the progress of social evolution with its artificial methods of dress and living the woman has been rendered less capable of spontaneous delivery by reason of anatomical and physiological defects which often render outside aid necessary to accomplish the delivery, and with this unnatural method of delivery came the plague of puerperal sepsis and also, because the various mechanical and surgical principles of labor were not as well understood by the doctor as by nature herself an appalling amount of preventable morbidity has occurred.

Dismissing from our consideration the question of restoring woman to her pristine ability of spontaneous delivery it is our purpose to discuss the methods by which we may best meet the conditions now existing, to the end that these anatomical and physiological abnormalities may be overcome with the least resultant injury to the patients, and it is with the development of our surgical knowledge and skill to their highest excellence that we will make our greatest advance.

Professor Duhrssen has said that "Advances in obstetrics are to be made only by its thorough saturation with surgical principles."

Technically, surgery has meant a therapy of a distinctly operative kind, but good surgery depends on good diagnosis and when the diagnosis has been correctly made skilful treatment achieves its greatest triumph; just so in obstetrics the correct diagnosis may be followed by skilful operation with a good result, in any event the final care of any obstetrical case is distinctly operative and is surgical in so far as it is anything but obstetrical.

Obstetrics may include at times, nearly every branch of medical science from physical diagnosis on through physiological chemistry and general medicine to the highest development of the surgical art; they may and do each have their fair place in the conduct of a case and each in its place is most essential but in the realm of surgery does obstetrics find its most valued assistant, and let me here emphasize the point that surgery is *only* the *assistant* and under no circumstances must it dominate the field.

You cannot take from woman, entirely, her function of child bearing and transfer it to the operating table, but as we have noted, there is an increasing proportion of cases abnormal and it is in these cases where good diagnostic and surgical skill will accomplish wonderful results in the preservation of the life and future health of the patients.

The knowledge of bacteriology and the value of strict asepsis in obstetrical work is well known to all and yet it is the trained surgeon who really understands it and who is able to give his patient the real benefit of our knowledge of asepsis or can give the best results when it is necessary to go into the field of antisepsis.

The foregoing remarks have their application to the whole field of obstetrical work, the easy normal case as well as the more difficult, but with particular emphasis do they obtain in those abnormal cases which require definite outside aid to accomplish the delivery and in which there is such an element of danger of infection from that cause.

Let us consider briefly some of the most prominent of the surgical operations that have done so much to rid these desperate cases of much of their dread mortal and morbid results.

The use of forceps of one kind or another to aid in the delivery dates from very early times and has been, perhaps, the most beneficent surgical procedure that has ever been adopted for the relief of suffering, but at the same time it has been the source of untold damage to many patients and I cannot make my language too strong to impress it upon you that the obstetrical forceps, in unskilled hands and in the face of dystocia has possibilities of the direst injury and let me urge upon you that unless you are perfectly sure of your diagnosis of the child's position do not apply the forceps. Illustration: A vertex presentation can be delivered face to the pubis but it is usually a crime to do it and if it is done it will be at the expense of the pelvic floor.

Scientific application of the forceps in moderate degrees of dystocia and under conditions of the most rigid surgical asepsis is a most laudable surgical procedure and should be resorted to more often by men who are familiar with good surgical technic and obstetrical mechanics.

The application of forceps at or above the pelvic brim is a difficult and dangerous surgical operation and should be resorted to only subsequent to an accurate diagnosis and by one who is thoroughly familiar with the work and who appreciates the dangers and the difficulties to be overcome.

After the application of forceps the next surgical operation in the obstetrical field in importance and frequency of adoption is internal podalic version.

Version should be done in vertex cases with a floating head where rapid delivery is indicated, and sometimes in the presence of dystocia instead of high forceps. Perhaps its most frequent adoption is in cases of antepartum bleeding. It competes with both high forceps and Cesarean section and sometimes with hebotomy, or may possibly be used in connection with the vaginal cesarean.

Classical Cesarean section, elective, is rapidly becoming the operation of choice in difficult obstetrical work and the indications for its adoption are constantly broadening until now it is done for many lesser degrees of pelvic deformity than was formerly deemed necessary and it is also frequently employed in cases where a rapid delivery is imperative, such as placenta previa, eclampsia, etc. The writer feels very emphatically that an elective Cesarean done under strict surgical conditions is far safer than many of the operations for delivery through the pelvis.

Hebotomy or vaginal Cesarean is indicated with the woman in labor, the head in the pelvis and a condition developed which renders a rapid termination of the first stage of labor imperative. It was first suggested by Duhrssen and consisted of two incisions through the lower uterine segment, the application of forceps and an immediate suture following delivery. It has been modified now to one incision in the anterior median line after carefully separating the bladder from the cervix.

The old operation of symphyseotomy has been replaced by pubiotomy or section of the pubic bone just external to the pubic spine and has been proven to be more satisfactory than the older plan and is indicated in the later stages of labor with the head in the pelvis and a contracted outlet, or perhaps with the head at the brim of a moderately contracted pelvis, if there is a question of any infection of the patient.

666 East Avenue.

